# A Review of the Occurrence of Alpha-Emitting Radionuclides in Wild Mushrooms

**DOI:** 10.3390/ijerph17218220

**Published:** 2020-11-06

**Authors:** Dagmara Strumińska-Parulska, Jerzy Falandysz

**Affiliations:** 1 Toxicology and Radiation Protection Laboratory, Faculty of Chemistry, University of Gdańsk, 80-308 Gdańsk, Poland; 2 Environmental Chemistry & Ecotoxicology Laboratory, Faculty of Chemistry, University of Gdańsk, 80-308 Gdańsk, Poland; jerzy.falandysz@gmail.com; 3 Environmental and Computational Chemistry Group, School of Pharmaceutical Sciences, Zaragocilla Campus, University of Cartagena, Cartagena 130015, Colombia

**Keywords:** alpha-emitters, radioactivity, exposure, food, fungi, anthropogenic radionuclides, naturally occurring radionuclides

## Abstract

Alpha-emitting radioisotopes are the most toxic among all radionuclides. In particular, medium to long-lived isotopes of the heavier metals are of the greatest concern to human health and radiological safety. This review focuses on the most common alpha-emitting radionuclides of natural and anthropogenic origin in wild mushrooms from around the world. Mushrooms bio-accumulate a range of mineral ionic constituents and radioactive elements to different extents, and are therefore considered as suitable bio-indicators of environmental pollution. The available literature indicates that the natural radionuclide ^210^Po is accumulated at the highest levels (up to 22 kBq/kg dry weight (dw) in wild mushrooms from Finland), while among synthetic nuclides, the highest levels of up to 53.8 Bq/kg dw of ^239+240^Pu were reported in Ukrainian mushrooms. The capacity to retain the activity of individual nuclides varies between mushrooms, which is of particular interest for edible species that are consumed either locally or, in some cases, also traded on an international scale. The effective radiation dose from the ingestion of this food can reportedly range from 0.033 µSv/kg dw to 26.8 mSv/kg and varies depending on the country. Following pollution events, such consumption may expose consumers to highly radiotoxic decay particles produced by alpha emitters.

## 1. Introduction

Mushrooms are a highly biodiverse group of organisms, a part of the traditional gastronomic heritage of the world, and also an important source of nutrients for small and large wildlife [1]. Some mushrooms are seen as having healing properties and, above all, many, when well-prepared, have a delicious taste [2]. Mushrooms typically grow in forests and fields, but almost all ecosystems will support their growth in the proper substrate medium [3]. These fruiting bodies of fungi are relatively rich in minerals and trace metals (including radionuclides) on a dry weight (dw) basis [4,5,6].

Although the phenomenon of radioactivity was discovered over 100 years ago, the special significance recorded in the pages of radiochemistry and nuclear chemistry history was realized from the 1940s to the 1960s [7,8,9]. The development and use of nuclear energy and the testing of nuclear weapons have in the past created a flux of released artificial radiation emitters that have been deposited in various environmental and food compartments, undergone biogeochemical cycles, and thus necessitate the need for control and surveillance measures [9,10,11,12]. In particular, nuclear weapons testing has led to massive contamination of nuclear test sites [13], and atmospheric detonations (1945–1980) and major accidents at nuclear power plants (i.e., Chernobyl in 1986 and Fukushima in 2011) have resulted in a substantial spread of radioactive isotopes all over the world. To some extent, emissions can also arise from current human activities such as mining, coal burning, and oil and gas exploration [14,15,16,17,18,19,20]. A range of edible mushrooms, including species foraged from the wild, are efficient bio-accumulators of various radioactive contaminants [21,22,23,24,25,26].

Most of the studies on radioactivity in edible mushrooms reported so far have been dedicated to the activity concentration of less hazardous gamma emitters (electromagnetic radiation), such as the artificial nuclides ^134/137^Cs, and the natural nuclide, ^40^K [3,23,27,28,29,30,31,32]. From a radio-toxicological point of view, the nuclides of the greatest importance are medium- and long-lived alpha-radioactive isotopes. Alpha decay (α-decay) is a form of decay where an atomic nucleus produces an alpha particle and changes into a different atomic nucleus. An alpha particle equals the ^4^He nucleus, containing two protons and two neutrons. Alpha particles have energy from 2 (^147^Sm) to 8.8 MeV (^212^Po), with a median of 5 MeV and a velocity of about 15,000 km/s. Because of their rather large mass and low speed, alpha particles interact with other atoms. The high mass and charge of alpha particles, in relation to other forms of nuclear radiation, give them greater ionizing power, but the penetration depth is much smaller [30]. Every significant alpha emitter present in the environment belongs to Group A (highly toxic radioisotopes) [33]. Thus, the ingestion of an alpha emitter and the resulting exposure to the internal organs of humans or animals could be of high concern when the source is contaminated food or feed [34,35]. At present, ^210^Po is judged as one of the most hazardous radionuclides. It is 10,000 times more toxic than hydrogen cyanide, and alongside the botulinum toxin, it is one of the most toxic substances ever known [36]. The use of ^210^Po as a poison to kill Alexander Litvinenko in 2006 increased interest in the radio-toxicological properties of this radioisotope as well as its occurrence in the environment and bioaccumulation in food products [37].

The analysis of alpha-emitting nuclides in mushrooms, foods and other biological materials is very laborious, highly time-consuming, and expensive, because of the complicated analytical procedures that are required for low or ultralow physical occurrence and the required sensitivity of detection, selectivity and accuracy of analytical equipment [38]. In consequence, the amount of information available on the occurrence of alpha emitters in environmental matrices including mushrooms is much lower in comparison to other radionuclides and especially, as mentioned, the gamma emitters.

The aim of this review is to present an overview on the occurrence and bio-concentration of alpha radioactivity in mushrooms worldwide along with consideration of the health aspects for human consumers.

## 2. Alpha Emitters in Mushrooms

Analytical data on the occurrence of the alpha emitters (^210^Po, ^222^Rn, ^226^Ra, ^228^Th, ^230^Th, ^232^Th, ^234^U, ^235^U, ^236^U, ^238^U, ^238^Pu, ^239^Pu, ^240^Pu, ^241^Am) in mushrooms were collected from the available literature (49 papers and reports) and are systematically presented in Table 1 and Table 2. In a few cases, the collected literature data on the radioactivity concentration were converted (using a consensus value of 10% dry matter relative to fresh mushrooms) with the aim of normalization, comparability and presentation of the results based on a dry weight (dw). Unlike certain other nutritional elements, alpha-emitting radionuclides are not essential for fungi, but they are absorbed by the mycelium, bio-accumulated in the fruiting bodies (mushrooms) and cycled in the food webs which extend to animals and humans. The risk from low-level radiation is still unknown and is based on the linear hypothesis which has been in operation that demonstrated that the relationships between hazard and radiation dose at high levels of exposure also apply to low levels [33,39]. Baeza et al. analyzed ^239+240^Pu, ^234,238^U, ^228,230,232^Th, and ^226^Ra bioaccumulation and distribution in a series of cultures of the species *Pleurotus eryngii* under controlled laboratory conditions. During the growth period, ^226^Ra was located in the gills of the cap, while ^239+240^Pu, ^234,238^U, and ^228,230,232^Th were observed mostly in the remaining part of the cap [40]. However, there are no studies on inter-species differences in alpha-emitters accumulation in cultivated mushrooms under controlled conditions.

### 2.1. Naturally Occurring Radioisotopes in Mushrooms

Natural radiation includes permanent low-level cosmic radiation and the radiation arising from the decay of naturally occurring radionuclides, namely the primordial radioactive elements in the crust of the Earth and their radioactive decay products (natural radioactive decay chains). Thus, human exposure to radiation has always been an unavoidable effect of the ubiquitous distribution of natural radioactivity [41]. Cosmogenic radionuclides (i.e., ^3^H, ^14^C) are produced constantly by a bombardment of stable nuclides by cosmic rays, principally in the atmosphere. The origin of the primordial natural radionuclides of the Earth (i.e., ^40^K, ^87^Rb, ^235^U, ^238^U, ^232^Th) is connected to the phenomenon of nucleosynthesis in stars, and their half-lives are longer or comparable with the age of the Earth. The secondary natural radioactive elements found in the environment are directly joined to the very long half-lives of the parents of these chains: the uranium (parent nuclide ^238^U), thorium (parent nuclide ^232^Th), and actinium (parent nuclide ^235^U) decay chains [42]. 

Technologically enhanced naturally occurring radioactive materials (TENORM) consist of materials including, usually, industrial wastes or by-products enriched with radioactive elements found in the environment, such as uranium (U), thorium (Th), and potassium (K) and any of their decay products, such as radium (Ra) and radon (Rn), polonium (Po) and radiolead (Pb) [14,43,44,45,46]. The presence of nuclides in mushrooms and plant-based foods depends on the geological structure of the lithosphere, the agronomic condition of arable soils, and the climate [47,48]. In general, higher activity concentrations of radionuclides have been measured in Ramsar (Iran), Kerala and Madras (India), Yangjiang (China), Pakistan, Brazil and Sudan, in high natural radioactivity background areas or impacted by TENORM [15,47,49,50,51,52,53]. 

Activity concentrations of naturally occurring radionuclides accumulated in mushrooms worldwide vary over a wide range—about eight orders of magnitude (Table 1). The highest activity concentration among all the mushrooms studied so far has been reported for polonium ^210^Po (T_1/2_ = 138.4 days) that appears at the end of the decay chain of uranium ^238^U and is an interesting natural element to investigate due to its radioecology (high bioconcentration factor) and one of the highest radiotoxic characteristics [48,54]. ^210^Po enters the biosphere through various routes of terrestrial and marine radio-ecological pathways. The major source of contamination of flora and fauna with ^210^Po basically comes from contaminated soil or through aerosol-associated fallout from the atmosphere [48,55,56]. ^210^Po is more easily accumulated by mushrooms and has higher bioconcentration factors when compared to other alpha emitters [57]. The increase in polonium concentration in mushrooms may also be related to the chemical similarities with sulfur or selenium elements [58]. Polonium is an element from the oxygen family, together with sulfur, selenium and tellurium—so called chalcogen elements. The susceptibility of fungi to higher accumulation of ^210^Po than other alpha emitters could be explained in part by the type and quantity of sulfur (S) ligands that they produce, but this has not been studied so far. Mushrooms differ in their contents of sulfur, which is a major chemical element in mushrooms as well as the main element in ligands for, e.g., Hg or Se. The type and quantity of sulfur ligands associated with ^210^Po that bio-accumulate in mushrooms is a species-specific feature that is dependent on environmental conditions related to the soil bedrock background composition as well as anthropogenic pollution, as in the case of several other metallic elements [54].

The highest activity concentrations of ^210^Po have been reported in mushrooms from Scandinavia [23,59,60] and in mushrooms growing close to a uranium mine in Germany [61], while the lowest levels are seen in collections from Poland and New Zealand [57,62,63,64,65,66] (Table 1). The authors Guillén and Baeza (2014) noted that the pattern distribution of ^210^Po seemed to be species-dependent [67], but a comparison of inter-genus data shows that the most important aspects are local conditions, e.g., natural radioactivity, atmospheric fallout, geological conditions, etc. [20,23,57,59,61,63].

Radium, ^226^Ra (T_1/2_ = 1600 years), along with ^210^Po, belongs to the natural series of ^238^U [48]. The highest ^226^Ra activity concentrations have been reported in mushrooms collected in the vicinity of uranium mines or places with a higher natural uranium background [61,68,69]. The variation of ^226^Ra occurrence in mushrooms is substantially lower in the areas not affected by higher ^210^Po and ^226^Ra backgrounds [61,70,71,72,73,74,75,76,77,78,79] (Table 1). 

The uptake of thorium (^228^Th T_1/2_ = 1.91 years; ^230^Th T_1/2_ = 7.54 × 10^4^ years; ^232^Th T_1/2_ = 1.4 × 10^10^ years) by mushrooms, as well as uranium (^238^U T_1/2_ = 4.47 × 10^9^ years; ^235^U T_1/2_ = 7.04 × 10^8^ years; ^234^U T_1/2_ = 2.45 × 10^5^ years) [80,81] is lower than that of ^210^Po and ^226^Ra (Table 1), and this can be related to low bio-concentration factors for Th and U, regardless of their abundance in soil [4,5,82]. However, significant differences between uranium and thorium activity concentrations have been reported. In the case of thorium, the highest activity concentrations of ^228^Th and ^232^Th have been noticed in Brazil, with the lowest in Poland [73,76,78,83,84,85,86,87,88,89,90,91]. In the case of uranium, the highest activity concentrations of the alpha emitters ^234^U, ^235^U and ^238^U have been reported in mushrooms from Germany, Turkey, Finland, and Slovakia [60,61,86,88,90], with the lowest being in Poland, Serbia, Bosnia and Herzegovina as well as China (Yunnan) and New Zealand [58,62,70,71,78,83,84,89,91,92,93,94,95]. If the uranium activity is compared to its daughter nuclide, ^226^Ra, the maximum radium activity concentration is always higher than the activity of the parent (^238^U). This might suggest that they are not in equilibrium in mushrooms. The increase in radium activity concentration might be due to the chemical similarities with calcium, in much the same way as polonium is physically and chemically an analog of the chalcogen elements (group 16 of the periodic table) [58,67,96]. However, the reported data have shown that mushrooms may bioaccumulate ^234^U and ^238^U more effectively than ^230^Th and ^232^Th, while both elements (U and Th) were bio-excluded (bioaccumulation factor values in the range from 0.005 to 0.19) [86,89,91].

### 2.2. Anthropogenic (Artificial, Man-Made) Radioisotopes in Mushrooms

Anthropogenic radioactive contamination of the environment became a reality on 16 July 1945, when the first fission weapon was tested near the town of Alamogordo (New Mexico, USA). Nuclear weapon testing and accidents in civil installations have led to massive artificial (man-made) radioactive pollution that has been spread all over the world. Since 1945, at least eight nations have detonated 2047 nuclear devices, with between 423 and 520 being carried out in the atmosphere [19]. The production of plutonium and other transuranic isotopes has been estimated at 0.33 PBq of ^238^Pu, 7.8 PBq of ^239^Pu, 5.2 PBq of ^240^Pu, 170 PBq of ^241^Pu, 0.00037 PBq of ^241^Am, and 0.00026 PBq of ^244^Cm. In Europe, the nuclear accident at the Chernobyl Nuclear Power Plant caused the biggest radiation pollution event in the history of nuclear energy [101,102]. The incident released 20 kg of plutonium (0.025 PBq of ^238^Pu, 0.055 PBq of ^239,240^Pu, 5 PBq of ^241^Pu), 0.006 PBq of ^241^Am, and 0.006 PBq of ^243,244^Cm [9,103,104,105]. The most recent nuclear accident, namely at the Fukushima Daiichi nuclear power plant (NPP) was estimated to be 15 times smaller than that at Chernobyl [102]. It has released about 2.4–19 GBq of ^238^Pu, 0.41–3.2 GBq of ^239^Pu, 0.51–3.2 GBq of ^240^Pu and 9.8–100 GBq of ^242^Cm [106]. Since 2015, the atmospheric releases from the Fukushima Daiichi NPP have continued, but at very low levels which were not of radiological concern [106]. Thus, the medium- and long-lived radioactive isotopes and especially the alpha-isotopes (such as ^238^Pu, ^239^Pu, ^240^Pu, ^241^Am, ^243,244^Cm, ^237^Np) are of the greatest importance from the point of view of human health and functioning of ecosystems, as they are radioactive and toxic metals, and hazardous environmental pollutants [54,107].

Also activity concentrations of artificial radionuclides accumulated in mushrooms worldwide vary over a wide range (Table 2), but their reported range of contamination of mushrooms is smaller when compared to natural radioisotopes (Table 1 and Table 2). Nevertheless, the presence of man-made nuclides in mushrooms and plant-based foods depends on local radioactive pollution conditions (nuclear test sites, facilities, accidents and distance from these sites) and is influenced by global atmospheric fallout [9,12,108,109]. Due to analytical difficulties, very few studies have dealt with anthropogenic alpha emitters released into the environment, and have instead focused on the most common radioactive elements, namely plutonium and americium, as well as uranium ^236^U [83,84,85,86,92,108,110,111,112,113,114,115,116,117,118].

There is only one article on ^236^U (T_1/2_ = 2.34 × 10^7^ years) activity concentration in mushrooms [92], which is an activation product of ^235^U or a decay product of ^240^Pu. Its presence has been reported due to work on depleted uranium dispersion and pollution in the environment of the Balkan region as a result of the Balkan (Kosovo) War in 1999. The ^236^U activity concentrations in mushrooms determined by Jia et al. 2004 [92] ranged from 0.014 to 0.038 Bq/kg dw (Table 2). Among the four major plutonium isotopes, three are alpha emitters: ^238^Pu (T_1/2_ = 87.7 years), the fissile and the most important isotope, ^239^Pu (T_1/2_ = 24,110 years), as well as ^240^Pu, which is produced via neutron capture of ^239^Pu (T_1/2_ = 6564 years) [107]. Plutonium is not effectively accumulated by mushrooms and shows a similar distribution in fruiting bodies to that of thorium and uranium (stem > cap > gills) [40]. Baeza et al. studied ^239+240^Pu accumulation in the saprophytic fungus *Pleurotus eryngii* cultivated under laboratory conditions and its occurrence in wild-growing mycorrhizal mushroom species (*Tricholoma equestre*), and concluded that its distribution seemed to be species-dependent, but were not able to confirm whether saprophytic fungi presented a lower content of this radionuclide than mycorrhizal fungi, as was observed in the case of ^137^Cs [40,115]. The highest activity concentrations of Pu isotopes have been determined in mushrooms collected at sites in the Ukraine that were associated with indirect contamination from the Chernobyl incident fallout in 1986 [83,108,112]. Mushrooms of various species foraged elsewhere in Europe were affected by both sources—namely, the global atmospheric fallout from earlier weapons testing and the incident at Chernobyl [84,85,86,110,111,112,113,114,116,117,118] (Table 2).

The element americium may originate from global atmospheric fallout connected to nuclear weapon testing or as a decay product of its parent nuclide, ^241^Pu, which was identified in Chernobyl fallout [103]. So far, its presence has been measured and reported only in mushrooms sampled in European countries such as Finland, Slovakia, Spain and the UK, and the activity concentrations were the lowest of all the alpha emitters, i.e., in the range from 0.003 to 1.01 Bq/kg dw [86,113,114,115,116,117,118] (Table 2). 

Radioactive elements, especially alpha emitters, are not essential metals for biota. Due to their occurrence in the environment, they are bio-accumulated along with essential micronutrients as well as toxic elements. When compared to the essential trace elements, the activity concentrations of the alpha-emitting radionuclides in wild-growing mushrooms from unpolluted areas in this study were much lower (e.g., alpha emitters of U, Th, Ra, Rn) or lower (e.g., alpha emitters of Po, Pu, Am, or ^236^U) [5,91,119,120]. In some species, such as *Amanita citrina, Laccaria* sp., *Tricholoma populinum, Strobilomyces strobilaceus, Russula exalbicans, Leccinum aurantiacum, Hebeloma sinapizans,* and *Cantharellus cibarius*, the amounts of uranium and thorium were higher than stable lead (Pb) or silver (Ag) [4]. As mentioned, the occurrence of natural alpha-nuclides in mushrooms is related to the geochemistry of soil bedrock, agronomic activities and climate and the presence of man-made nuclides in mushrooms depends on local radioactive pollution (nuclear test sites, facilities or accidents as well as the distance from the accident sites) as well as the impact of global atmospheric fallout. If the location in which mushrooms are collected is contaminated with radioactive substances (e.g., Chernobyl or Fukushima area, nuclear test sites), the number of alpha emitters (especially transuranic, as ^235^U, ^236^U, ^238^U, ^239^Pu, ^240^Pu) might be higher than stable toxic trace metals, e.g., lead (Pb), mercury, (Hg) [13,47,83,113,121,122,123]. 

## 3. Risk Assessment

Humans are exposed to radiation from two types of sources: external, which includes radionuclides in the Earth and cosmic radiation, and internal radiation from radionuclides incorporated in the body. In this study, the key pathways of radionuclide intake are inhalation, and ingestion of food and water [124]. In the International System of Units (SI), the unit of ionizing radiation dose and a measurement of the biological health effect of ionizing radiation on the human body is the Sievert (Sv). If the dose is received over a very short duration, the LD_50/60_ is estimated at 4–5 Sv [125], so SI prefixes are frequently used: i.e., the millisievert (mSv) and microsievert (μSv). The assessment of the potential risk to human health connected to all ionizing radiation sources is phrased as the sum of all evaluated effective doses (E) from all sources (internal and external) and is mostly given in mSv per year. For exposure to the general public, the ICRP recommends that the limit should be expressed as an effective dose of 1 mSv annually [125]. An impact of the total annual effective dose on an adult from any food consumption may be presented as a dose from the mass of the food consumed (Sv/kg dw) and calculated as the function of the product of the radioisotope conversion coefficient (Sv/Bq) and its activity concentration (Bq/kg dw) in the foodstuff:

E = A × d_c_;

where:

A—activity concentration (Bq/kg dw),
d_c_—dose coefficient (conversion factor) (Sv/Bq), defined as the dose received from the unit of radioactivity intake; the ICRP conversion coefficients recommended for the ingestion of alpha emitters presented in the review in the case of adult members of the public, range from 4.5 × 10^−8^ Sv/Bq for ^238^U to 1.2 × 10^−6^ Sv/Bq for ^210^Po, with an average value of 10^−7^ Sv/Bq (exact values are shown in Table 1 and Table 2) [97].

The potential health hazard risk due to the consumption of mushrooms is presented in Table 1 and Table 2. These radioelements are toxic to humans, both chemically (connected to their toxic heavy metals properties; some with characteristics that are similar to the alkaline earth metal ions) and radiologically (connected to their nuclear disintegration), under certain conditions, and their toxicity varies depending on the chemical and physical forms, amounts ingested and route of exposure. If the exposure involves multiple active radioisotopes, the chemical and radiological toxicity can be additive or may potentiate in some instances [126,127,128]. However, the dose calculations for human radiological protection are very conservative and consider worst-case scenarios [29,97]. ^210^Po is one of the most important radionuclides to which humans are exposed [129]. ^210^Po, together with gaseous radon (^220^Rn, ^222^Rn), ^226^Ra, ^210^Pb, and ^40^K, are natural radioactive materials that deliver the highest dose to living organisms [43]. The associated radiation produces the same primary basic physicochemical effects of excitation and ionization within the biological material and differs only in the spatial distribution and intensity of these effects. At low levels of radiation, the clinical symptoms might not be observed (radiolysis and small genetic reactions: mutations, cells necrosis or apoptosis; changes in the blood, etc.) or might occur after a number of years as stochastic effects (i.e., leukemia). At much higher levels (more than 1 Sv), there are many observed (determined) symptoms depending on the magnitude of the dose: nausea and vomiting, headache, fatigue, fever, low number of white blood cells, bleeding, anemia, etc. [33,130]. 

Of the reported activity concentrations for mushrooms worldwide, the highest effective doses have been calculated for ^210^Po as a result of its high concentrations and the high value of its conversion coefficient. Thus, ^210^Po occurrence would be the main source of alpha radiation for mushroom consumers (0.28–26,400 µSv/kg dw) (Table 1). It is followed by ^226^Ra, which, at high concentrations, results in high effective doses for consumers (0.006–196 µSv/kg dw) (Table 1). The values of the annual radiation dose from thorium and uranium alpha emitters decay ingested through mushroom consumption may be much lower because these radionuclides are accumulated to a much lower extent. Although thorium radioisotopes are characterized by higher values of the conversion coefficient, the doses from their decay are comparable to uranium, because thorium is weakly accumulated (Table 1). The doses from anthropogenic alpha emitters have been significantly lower, even when mushrooms have been collected from potentially polluted areas (e.g., Ukraine) (Table 2). 

The total effective dose that might be received from the reported content of naturally occurring alpha-emitters has been estimated to be in the range of 0.007 µSv/kg dw–26.7 mSv/kg dw depending on the country (Table 1). Anthropogenic radionuclides might give an effective dose from 0.026 µSv/kg dw to 44.4 µSv/kg dw (Table 2). Thus, the total effective dose from all reported sources might in be the range of 0.033 µSv/kg dw to 26.8 mSv/kg dw. As mentioned before, the recommended yearly adult dose limit is 1 mSv for the general public [97]. Depending on the origin of the wild mushroom, some specimens may be highly enriched with alpha-emitting radionuclides and the resulting effective dose may be significantly higher than permitted values. In unpolluted areas, the main source of radiation would be naturally occurring radionuclides, especially ^210^Po and ^226^Ra.

## 4. Conclusions

The main pathways of radionuclide exposure are ingestion of food and water and inhalation. Among food products, wild mushrooms are a possible source of radionuclides as they accumulate radioactive elements in much the same way as other metals, and various species have different retention capacities for individual radionuclides. The radioactive analogs of essential elements are effectively accumulated, i.e., ^210^Po and ^226^Ra, while other heavy radioelements (especially artificial radionuclides) are not heavily accumulated. Thus, naturally occurring radionuclides are the most abundantly occurring of all the bio-accumulated alpha emitters. Local geological conditions and potential radioactive pollution are the most important factors influencing the bioaccumulation level. Thus, depending on the origin of the mushrooms, some might be highly enriched with alpha-emitting radionuclides, and the effective dose might be significantly higher than suggested values (i.e., IAEA, ICRP). In unpolluted areas, the main source of radiation would be naturally occurring radionuclides, especially ^210^Po and ^226^Ra.

## Figures and Tables

**Table 1:** The activity concentration ranges of naturally occurring alpha-emitting radioisotopes in wild-growing mushrooms (the Latin names of the species are cited as described by the authors—the current names of some species can be found on the Index Fungorum web site) collected from different countries as well as the effective dose from mushrooms consumption presented as a proportion of total dietary consumption (µSv/kg dw) (* calculation based on the isotopic activity concentration and effective dose coefficients (Sv/Bq) for ingestion of radionuclides for adults [97]).

Radionuclide (Sv/Bq)	Species	Activity Concentration (Bq/kg dw)	Effective Dose * (µSv/kg dw)	Country	References
^210^Po (1.2 × 10^−6^)	*Baorangia bicolor*, *Boletus bainiugan*, *B. calopus*, *B. flammans*, *B. obsclereumbrinus*, *Butyriboletus roseoflavus*, *Rubroboletus sinicus*, *Rugiboletus extremiorientale*, *Wolfiporia cocos*	1.66–308	1.99–370	China	[58,98,99]
	*Albatrellus ovinus*, *Cantharellus cibarius*, *Cortinarius armillatus*, *C. caperatus*, *Craterellus cornucopioides*, *C. tubaeformis*, *Hygrophorus camarophyllus*, *Lactarius rufus*, *L. scrobiculatus*, *L. utilis*, *L. torminosus*, *Leccinum variicolor*, *L. versipelle*, *L. vulpinum*, *Rozites caperatus*, *Russula aeruginea*, *R. decolorans*, *R. paludosa*, *R. parazurea*, *R. xerampelina*, *R. vinosa*, *Suillus luteus*	6–22,000	7.2–26,400	Finland	[59,60]
	*Agaricus* sp., *Boletus* sp., *Leccinum* sp., *Lepiota* sp., *Lycoperda* sp., *Suillus* sp., *Xerocomus* sp.	1.0–640	1.2–768	Germany	[61]
	Not specified	<9	<10.8	New Zealand	[62]
	*Leccinum scabrum*, *L. versipelle*, *Russula paludosa*, *R. decolorans*	4.7–198	5.64–238	Norway	[23]
	*Amanita muscaria*, *A. rubescens*, *A. phalloides*, *Agaricus silvicolae-similis*, *Boletus edulis*, *Cantharellus cibarius*, *Hydnum repandum*, *Imleria badia*, *Lactarius torminosus*, *Leccinum scabrum*, *L. versipelle*, *L. aurantiacum*, *L. aurantiacum var. quercinum*, *L. vulpinum*, *L. aurantiacum var. duriusculum*, *Leccinellum pseudoscabrum*, *Lycoperdon excipuliforme*, *Macrolepiota procera*, *Marasmius oreades*, *Russula cyanoxantha*, *R. nobilis*, *R. solaris*, *Scleroderma citrinum*, *Strobilomyces strobilaceus*, *Tylopilus felleus*, *Suillus bovinus*, *S. luteus*, *Xerocomus badius*, *X. subtomentosus*	0.23–17	0.28–20.4	Poland	[57,63,64,65,66,100]
^222^Rn	*Boletus edulis*, *Leccinum aurantiacum*, *L. scabrum*, *Paxillus involutus*	16–36	-	Russia	[78]
^226^Ra (2.8 × 10^−7^)	*Amanita fulva*, *Armillaria mellea*, *Boletus edulis*, *Cantharellus cibarius*, *Flammulina velutipes*, *Gomphus clavatus*, *Hydnum repandum*, *Leccinum scabrum*, *Macrolepiota procera*, *Paxillus involutus*, *Rozites caperata*, *Russula cyanoxantha*, *R. emetica*, *Suillus variegatus*, *Xerocomus badius*	22–48	6.16–13.4	Austria	[70]
	*Agaricus campestris*, *A. blazei*, *Agaricus* sp., *Lentinula edodes*, *Pleurotus eryngii*, *P. ostreatus*, *P. ostreatoroseus*	7.3–66	2.04–18.5	Brazil	[74]
	*Agaricus blazei*, *Armillaria solidipes*, *Boletus aereus*, *B. brunneissimus, B. edulis*, *Boletus* sp., *Cantharellus cibarius*, *Cyclocybe parasitica*, *Flammulina velutipes*, *Gomphus floccosus, Hygrophorus russula*, *Hypsizygus ulmarius*, *Lactarius hatsudake*, *L. volemus*, *Lentinula edodes*, *Leucocalocybe mongolica*, *Macrolepiota albuminosa*, *Neoboletus obscureumbrinus*, *Pleurotus djamor*, *Termitomyces albuminosus*, *Tylopilus balloui*, *T. felleus*	0.05–3.65	0.014–1.02	China	[79]
	*Terfezia* sp.	419	117	Egypt	[68]
	Not specified	29.3–61.6	8.20–17.3	France	[71]
	*Agaricus* sp., *Boletus* sp., *Leccinum* sp., *Lepiota* sp., *Lycoperdon* sp., *Suillus* sp., *Xerocomus* sp.	0.3–512	0.084–143	Germany	[61]
	*Agaricus campestris*, *A. xanthodermus*, *Armillaria mellea*, *Boletus* sp., *Bovista plumbea*, *Chroogomphus rutilus*, *Clavariadelphus truncates*, *Clitocybe nebularis*, *Clitocybe* sp., *Cortinarius* sp., *Entoloma* sp., *Hydnellum concrescens*, *Hydnum rufescens*, *Hygrocybe acutoconica*, *Inocybe* sp., *Lactarius salmonicolor*, *Lepista flaccida*, *L. nuda*, *Lycoperdon perlatum*, *Macrolepiota mastoidea*, *Marasmius oreades*, *Melanoleuca cognata*, *Mycena seynii*, *Ramaria formosa*, *R. obtusissima*, *Russula delica*, *Sarcodon martioflavus*, *Suillus collinitus*, *Vascellum pratense*	0.3–1.0	0.084–0.28	Greece	[77]
	*Terfezia* sp., and not specified edible mushrooms	60–700	16.8–196	Iran	[68,69]
	*Terfezia* sp.	439	122	Kuwait	[68]
	*Pleurotus squarrosulus*, *Psathyrella atroumbonata*, *Pleurotus tuber-regium*, *Termitomyces striatus*, *T. robustus*	2.68–21.6	0.75–6.05	Nigeria	[76]
	*Boletus edulis*, *Leccinum aurantiacum*, *L. scabrum*, *Paxillus involutus*	29–78	8.12–21.8	Russia	[78]
	*Amanita muscaria*, *A. curtipes*, *Clitocybe* sp., *Gymnopilus penetrans*, *Hebeloma cylindrosporum*, *Lactarius deliciosus*, *Lycoperdon perlatum*, *Plerurotus eryngii*, *Rhizopogon roseolus*, *Russula cessans*, *R. toruosa*, *Tricholoma equestre*, *T. pessandatum*, *T. terreum*	0.021–62	0.006–17.4	Spain	[40,72]
	*Boletus* sp., *Brunneoporus malicola*, *Fomitopsis pinicola*, *Ganoderma applanatum*, *Hericium clathroides*, *Megacollybia platyphylla*, *Pluteus cervinus*, *Suillellus luridus*	4–14	1.12–3.92	Serbia	[75]
	*Terfezia* sp.	438	122	Tunisia	[68]
	*Agaricus campestris*, *Agaricus porphyrocephalus*, *Boletus edulis*, *Craterellus cornucopioides*, *Cantharellus cibarius*, *Lepiota cristata*, *Lycogala epidendrum*, *Marasmius oreades*, *Morchella esculenta*, *Nectria cinnabarina*, *Stropharia coronilla*	4.4–5.2	1.23–1.46	Turkey	[73]
^228^Th (7.2 × 10^−8^)	Not specified	3.1–127	0.22–9.14	Brazil	[87]
	*Armillaria mellea*, *Boletus reticulatus*, *Cantharellus cibarius*, *Grifola frondosa*, *Lactarius deliciosus*, *Leccinum* sp., *Suillus luteus*	0.34–31.8	0.025–2.29	Slovakia	[86]
	*Amanita muscaria*, *A. ponderosa*, *Hebeloma cylindrosporum*, *Lactarius deliciosus*, *Macrolepiota procera*, *Plerurotus eryngii*, *Rhizopogon roseolus*, *Russula cessans*, *Suillus bovinus*, *Terfezia arenaria*, *T. boudieri*, *Tricholoma equestre*, *T. terreum*, *Tricholoma* sp.	1.4–13	0.11–0.94	Spain	[40,84,85]
^230^Th (2.1 × 10^−7^)	*Leccinellum pseudoscabrum*, *Leccinum aurantiacum*, *L. aurantiacum var. duriusculum*, *L. aurantiacum var. quercinum*, *L. vulpinum*	0.04–2.13	0.001–0.16	Poland	[91]
	*Cantharellus cibarius*, *Grifola frondosa*, *Lactarius deliciosus*, *Leccinum* sp., *Suillus luteus*	0.05–3.75	0.004–0.27	Slovakia	[86]
	*Agaricus campestris*, *Amanita muscaria*, *A. ponderosa*, *Hebeloma cylindrosporum*, *Lactarius deliciosus*, *Macrolepiota procera*, *Omphalotus olearius*, *Plerurotus eryngii*, *Rhizopogon roseolus*, *Russula cessans*, *Suillus bovinus*, *Terfezia arenaria*, *T. boudieri*, *Tricholoma equestre*, *T. terreum*, *Tricholoma* sp.	0.053–6.9	0.004–0.50	Spain	[40,83,84,85]
^232^Th (2.3 × 10^−7^)	Not specified	0.6–142	0.14–32.7	Brazil	[87]
	*Terfezia* sp.	1.76–3.71	0.41–0.85	Iraq	[89]
	*Pleurotus squarrosulus*, *Psathyrella atroumbonata*, *Pleurotus tuber-regium*, *Termitomyces striatus*, *T. robustus*	8.57–14.3	1.97–3.29	Nigeria	[76]
	*Leccinellum pseudoscabrum*, *Leccinum aurantiacum*, *L. aurantiacum var. duriusculum*, *L. aurantiacum var. quercinum*, *L. vulpinum*	0.02–0.63	0.005–0.15	Poland	[91]
	*Boletus edulis*, *Leccinum aurantiacum*, *L. scabrum*, *Paxillus involutus*	13–33	2.99–7.59	Russia	[78]
	*Armillaria mellea*, *Boletus reticulatus*, *Cantharellus cibarius*, *Grifola frondosa*, *Lactarius deliciosus*, *Leccinum* sp., *Suillus luteus*	0.04–4.59	0.009–1.06	Slovakia	[86]
	*Agaricus campestris*, *Amanita muscaria*, *A. ponderosa*, *Hebeloma cylindrosporum*, *Lactarius deliciosus*, *Macrolepiota procera*, *Omphalotus olearius*, *Plerurotus eryngii*, *Rhizopogon roseolus*, *Russula cessans*, *Suillus bovinus*, *Terfezia arenaria*, *T. boudieri*, *Tricholoma equestre*, *T. terreum*, *Tricholoma* sp.	0.061–10.7	0.014–2.46	Spain	[40,83,84,85]
	*Agaricus campestris*, *A. porphyrocephalus*, *Amanita rubescens*, *Boletus edulis*, *Bonomyces sinopicus, Cantharellus cibarius*, *Craterellus cornucopioides*, *C. lutescens*, *Hygrophoropsis aurantiaca*, *Hypholoma fasciculare*, *Hypholoma* spp., *Lepiota cristata*, *Lycogala epidendrum, Marasmius oreades*, *Morchella esculenta*, *Nectria cinnabarina*, *Paxillus involutus, Pleurotus cornucopiae*, *Pycnoporus cinnabarinus*, *Pycnoporus* spp., *Russula delica, Stropharia coronilla*	0.35–182	0.081–41.9	Turkey	[73,88,90]
^234^U (4.9 × 10^−8^)	Not specified	0.26	0.013	Bosnia and Herzegovina	[56]
	*Boletus bainiugan*	0.19–0.89	0.009–0.044	China	[58]
	*Fomes fomentarius*	1.0–6.90	0.049–0.34	Kosovo	[92,95]
	Not specified	<5	<0.24	New Zealand	[62]
	*Armillaria mellea*, *Boletus edulis*, *Lactifluus vellereus*, *Leccinellum pseudoscabrum*, *Leccinum aurantiacum*, *L. aurantiacum var. duriusculum*, *L. aurantiacum var. quercinum*, *L. vulpinum*, *Macrolepiota procera*, *Xerocomus badius*	0.014–0.43	0.001–0.021	Poland	[83,91]
	Not specified	0.48–0.80	0.023–0.039	Serbia	[93]
	*Armillaria mellea*, *Boletus reticulatus*, *Cantharellus cibarius*, *Grifola frondosa*, *Lactarius deliciosus*, *Leccinum* sp., *Suillus luteus*	0.46–86.3	0.022–4.23	Slovakia	[86]
	*Agaricus campestris*, *Amanita muscaria*, *A. ponderosa*, *Hebeloma cylindrosporum*, *Lactarius deliciosus*, *Macrolepiota procera*, *Omphalotus olearius*, *Pleurotus eryngii*, *Rhizopogon roseolus*, *Russula cessans*, *Suillus bovinus*, *Terfezia arenaria*, *Terfezia boudieri*, *Tricholoma equestre*, *T. terreum*, *Tricholoma* sp.	0.15–7.0	0.007–0.34	Spain	[40,83,84,85]
^235^U (4.7 × 10^−8^)	Not specified	0.02	0.009	Bosnia and Herzegovina	[94]
	*Boletus bainiugan*	0.003–0.064	0.0001–0.003	China	[58]
	Not specified	1.56–5.61	0.073–0.27	France	[71]
	*Fomes fomentarius*	0.070–0.52	0.003–0.024	Kosovo	[92,95]
	*Armillaria mellea*, *Boletus edulis*, *Lactifluus vellereus*, *Macrolepiota procera*, *Xerocomus badius*	0.006–0.010	0.0003–0.0005	Poland	[83]
	Not specified	0.02–0.03	0.009–0.01	Serbia	[93]
	*Agaricus campestris*, *Amanita muscaria*, *A. ponderosa*, *Hebeloma cylindrosporum*, *Lactarius deliciosus*, *Macrolepiota procera*, *Omphalotus olearius*, *Plerurotus eryngii*, *Rhizopogon roseolus*, *Russula cessans*, *Suillus bovinus*, *Terfezia arenaria*, *T. boudieri*, *Tricholoma equestre*, *T. terreum*, *Tricholoma* sp.	0.007–0.42	0.0003–0.019	Spain	[40,83,84]
^238^U (4.5 × 10^−8^)	*Amanita fulva*, *Armillaria mellea*, *Boletus edulis*, *Cantharellus cibarius*, *Flammulina velutipes*, *Gomphus clavatus*, *Hydnum repandum*, *Leccinum scabrum*, *Macrolepiota procera*, *Paxillus involutus*, *Rozites caperata*, *Russula cyanoxantha*, *R. emetica*, *Suillus variegatus*, *Xerocomus badius*	44–92	1.98–4.14	Austria	[70]
	Not specified	0.27	0.013	Bosnia and Herzegovina	[94]
	*Agaricus blazei*, *Armillaria solidipes*, *Boletus aereus*, *B. bainiugan*, *B. brunneissimus*, *B. edulis*, *Boletus* sp., *Cantharellus cibarius*, *Cyclocybe parasitica*, *Flammulina velutipes*, *Gomphus floccosus*, *Hygrophorus russula*, *Hypsizygus ulmarius*, *Lactarius hatsudake*, *L. volemus*, *Lentinula edodes*, *Leucocalocybe mongolica*, *Macrolepiota albuminosa*, *Neoboletus obscureumbrinus*, *Pleurotus djamor*, *Termitomyces albuminosus*, *Tylopilus balloui*, *T. felleus*	0.15–7.68	0.007–0.34	China	[58,79]
	*Cantharellus cibarius*, *C. tubaeformis*, *Craterellus cornucopioides*, *Lactarius rufus*	92	4.14	Finland	[60]
	*Boletus* sp., *Suillus* sp., *Xerocomus* sp., *Leccinum* sp., *Lepiota* sp., *Agaricus* sp., *Lycoperdon* sp.	0.1–259	0.004–11.6	Germany	[61]
	*Terfezia* sp.	2.3–5.88	0.10–0.26	Iraq	[89]
	*Fomes fomentarius*	0.7–11.3	0.03–0.51	Kosovo	[92,95]
	Not specified	4	0.18	New Zealand	[62]
	*Armillaria mellea*, *Boletus edulis*, *Lactifluus vellereus*, *Leccinellum pseudoscabrum*, *Leccinum aurantiacum*, *L. aurantiacum var. duriusculum*, *L. aurantiacum var. quercinum*, *L. vulpinum*, *Macrolepiota procera*, *Xerocomus badius*	0.015–0.51	0.0007–0.023	Poland	[83,91]
	*Boletus edulis*, *Leccinum aurantiacum*, *L. scabrum*, *Paxillus involutus*	7.4–19	0.33–0.85	Russia	[78]
	Not specified	0.67–1.11	0.03–0.05	Serbia	[93]
	*Armillaria mellea*, *Boletus reticulatus*, *Cantharellus cibarius*, *Grifola frondosa*, *Lactarius deliciosus*, *Leccinum* sp., *Suillus luteus*	0.45–99.4	0.020–4.47	Slovakia	[86]
	*Agaricus campestris*, *Amanita muscaria*, *A. ponderosa*, *Hebeloma cylindrosporum*, *Lactarius deliciosus*, *Macrolepiota procera*, *Omphalotus olearius*, *Pleurotus eryngii*, *Rhizopogon roseolus*, *Russula cessans*, *Suillus bovinus*, *Terfezia arenaria*, *T. boudieri*, *Tricholoma equestre*, *T. terreum*, *Tricholoma* sp.	0.12–7.30	0.005–0.33	Spain	[40,83,84,85]
	*Amanita rubescens*, *Bonomyces sinopicus*, *Cantharellus cibarius*, *Craterellus lutescens*, *Hygrophoropsis aurantiaca*, *Hypholoma fasciculare*, *Hypholoma* spp., *Pycnoporus cinnabarinus*, *Pycnoporus* spp., *Paxillus involutus*, *Pleurotus cornucopiae*, *Russula delica*	1.03–168	0.046–7.56	Turkey	[88,90]

**Table 2:** The activity concentration ranges of anthropogenic alpha-emitting radioisotopes in wild-growing mushrooms (the Latin names of the species are cited as described by the authors—the current names of some species can be found on the Index Fungorum web site) collected from a different countries as well as the effective dose from mushrooms consumption presented as a proportion of total dietary consumption (µSv/kg dw) (* calculation based on the isotopic activity concentration and effective dose coefficients (Sv/Bq) for ingestion of radionuclides for adults [97]).

Radionuclide (Sv/Bq)	Species	Activity Concentration (Bq/kg dw)	Effective Dose * (µSv/kg dw)	Country	References
^236^U (4.7 × 10^−8^)	Not specified	0.014–0.038	0.0007–0.002	Kosovo	[92]
^238^Pu (2.3 × 10^−7^)	*Armillaria mellea*, *Boletus edulis*, *Lactifluus vellereus*, *Macrolepiota procera*, *Xerocomus badius*	0.0001–0.031	0.00002–0.007	Poland	[83]
	*Armillaria mellea*, *Boletus reticulatus*, *Cantharellus cibarius*, *Grifola frondosa*, *Lactarius deliciosus*, *Leccinum* sp., *Suillus luteus*	0.02–0.78	0.005–0.18	Slovakia	[86]
	*Agaricus campestris*, *Amanita muscaria*, *A. ponderosa*, *Hebeloma cylindrosporum*, *Lactarius deliciosus*, *Macrolepiota procera*, *Omphalotus olearius*, *Rhizopogon roseolus*, *Russula cessans*, *Suillus bovinus*, *Terfezia arenaria*, *T. boudieri*, *Tricholoma terreum*, *Tricholoma* sp.	0.0008–0.020	0.0002–0.005	Spain	[83,84]
	Not specified	0.024–0.09	0.005–0.027	UK	[117,118]
	*Boletus edulis*, *Cantharellus cibarius*, *Paxillus involutus*, *Suillus luteus*, *Xerocomus badius*	0.029–43.6	0.007–10.03	Ukraine	[83,108,112]
^239^Pu (2.5 × 10^−7^)	Not specified	0.1	0.025	UK	[113]
^239+240^Pu (2.5 × 10^−7^)	*Russula decolorans*	0.002–0.02	0.0005–0.005	Finland	[114,116]
	*Armillaria mellea*, *Boletus edulis*, *Lactifluus vellereus*, *Leccinum* sp., *Macrolepiota procera*, *Xerocomus badius*	0.001–0.09	0.0003–0.023	Poland	[110,111]
	*Armillaria mellea*, *Boletus reticulatus*, *Cantharellus cibarius*, *Grifola frondosa*, *Lactarius deliciosus*, *Leccinum* sp., *Suillus luteus*	0.07–3.16	0.017–0,79	Slovakia	[86]
	*Agaricus campestris*, *Amanita muscaria*, *A. ponderosa*, *Clitocybe* sp., *Hebeloma cylindrosporum*, *Lactarius deliciosus*, *Lycoperdon perlatum, Macrolepiota procera*, *Omphalotus olearius*, *Pleurotus eryngii*, *Rhizopogon roseolus*, *Russula cessans*, *Suillus bovinus*, *Terfezia arenaria*, *T. boudieri*, *Tricholoma equestre*, *T. terreum*, *Tricholoma* sp.	0.0066–0.246	0.0016–0.061	Spain	[40,83,84,115]
	Not specified	0.16–1.0	0.04–0.25	UK	[117,118]
	*Boletus edulis*, *Cantharellus cibarius*, *Paxillus involutus*, *Suillus luteus*, *Xerocomus badius*	0.053–53.78	0.013–13.45	Ukraine	[83,108,112]
^241^Am (2.0 × 10^−7^)	*Russula decolorans*	0.003–0.01	0.0006–0.002	Finland	[114,116]
	*Armillaria mellea*, *Boletus reticulatus*, *Cantharellus cibarius*, *Grifola frondosa*, *Lactarius deliciosus*, *Leccinum* sp., *Suillus luteus*	0.02–1.01	0.004–0.20	Slovakia	[86]
	*Amanita muscaria*, *Clitocybe* sp., *Hebeloma cylindrosporum*, *Lactarius deliciosus*, *Lycoperdon perlatum*, *Rhizopogon roseolus*	0.0086–0.067	0.0017–0.013	Spain	[115]
	Not specified	0.065–0.98	0.013–0.19	UK	[113,117,118]
